# Bowel preparation using 2-L split-dose polyethylene glycol regimen plus lubiprostone versus 4-L split-dose polyethylene glycol regimen: a randomized controlled trial

**DOI:** 10.1186/s12876-022-02497-2

**Published:** 2022-09-17

**Authors:** Apichet Sirinawasatien, Pallop Sakulthongthawin, Kanokpoj Chanpiwat, Tanyaporn Chantarojanasiri

**Affiliations:** 1 Division of Gastroenterology, Department of Medicine, Rajavithi Hospital, College of Medicine, Rangsit University, 2, Phayathai Road, Rajathewi, Bangkok, 10400 Thailand; 2 Department of Internal Medicine, Chaophraya Abhaibhubejhr Hospital, Tha Ngam Sub-District, Mueang Prachin Buri District, Prachin Buri 25000 Thailand

**Keywords:** Colonoscopy, Bowel preparation, Lubiprostone, Polyethylene glycol, Boston Bowel Preparation Scale

## Abstract

**Background:**

Colonoscopy is a standard procedure for evaluating colon diseases and screening for colorectal cancer, and bowel cleanliness prior to colonoscopy is key. The aim of this study was to compare the bowel cleansing efficacy of low-volume (2 L) split-dose polyethylene glycol (PEG) plus single-dose (24 µg) lubiprostone (LB) and high-volume (4 L) split-dose PEG.

**Methods:**

Patients scheduled to undergo outpatient colonoscopy between December 2019 and June 2021 at Rajavithi Hospital were enrolled and randomized into two groups: 2 L PEG + LB or 4 L PEG. Colon cleanliness was evaluated using the Boston Bowel Preparation Scale (BBPS) by reviewing images of the colon after completion of colonoscopy. Secondary outcomes comprised cecal intubation rate, procedure time, withdrawal time, polyp detection rate, adenoma detection rate, patient satisfaction, compliance (based on complete ingestion of bowel preparation regimen), willingness to repeat the preparation regimen, and associated adverse events.

**Results:**

One hundred and forty patients were included, with 70 in each group. The mean total and segment-specific BBPS scores were not significantly different between groups. However, the rate of adequate bowel preparation was significantly higher in the 2 L PEG + LB group than the 4 L PEG group (100% [95% CI 94.6–100] versus 88.4% [95% CI 78.4–94.9], *p* = 0.004) in the per-protocol analysis. Colonic polyps were the most common finding. The polyp detection rate, adenoma detection rate, and all secondary outcomes were statistically similar in the two groups (*p* > 0.05).

**Conclusions:**

The combination of 2 L split-dose PEG plus LB improves bowel cleanliness (based on BBPS scores) to a comparable degree to the standard 4 L split-dose PEG, without additional adverse events and with a lower PEG volume.

## Background

Colon disease assessment and colorectal cancer screening currently rely on colonoscopy as the standard approach. Clear mucosal visualization, which depends on the quality of the bowel preparation, is essential for detecting lesions and may contribute to the early detection of colorectal cancer [1]. Suboptimal preparation can hinder detection of smaller lesions, lengthen the procedure time, increase the risk of adverse events related to the procedure, and increase costs due to the need for repeated examinations and/or reducing the interval between surveillance examinations [2–4]. Meanwhile, 12.5–20% of exams involved inadequate bowel preparation. [1, 3]

Polyethylene glycol (PEG) is a balanced electrolyte solution that is associated with minimal water and electrolyte absorption or secretion during total gut perfusion [5]. It is currently the most commonly used solution for bowel preparation. However, the large volume of 4 Liter (L) required is poorly tolerated and can lead to nausea, cramping, and vomiting [6]. A pooled analysis of 15 trials found that 29% of patients were unable to completely ingest the PEG solution [7], which often results in unsuccessful bowel preparation and incomplete visualization. Low-volume PEG has been combined with stimulant laxatives (e.g., bisacodyl or senna) in order to improve bowel cleansing and reduce the volume of PEG required, but due to adverse events (including ischemic colitis caused by bisacodyl [8] and severe abdominal pain caused by senna), this combination is not widely used in general practice.

Lubiprostone (LB) is a locally acting activator of type 2 chloride channels in the gastrointestinal tract that enhances intestinal fluid secretion, resulting in softened stools and increased intestinal transit without the loss of either net intravascular fluid or electrolytes [9]. After oral administration of a single dose of 24 µg LB, the plasma concentration peaks within approximately 1 h, and the half-life of LB is approximately 3 h. [10] LB is currently approved by the US Food and Drug Administration (FDA) and Thailand FDA for the treatment of chronic idiopathic constipation, and it is generally well tolerated with an excellent safety profile.

The present study aimed to determine whether the efficiency of bowel cleansing with a combination of LB pretreatment and low-volume (2 L) split-dose PEG is equal to that of high-volume (4 L) split-dose PEG. Moreover, we aimed to compare cecal intubation rate, procedure time, withdrawal time, polyp detection rate, adenoma detection rate, patient satisfaction, compliance, willingness to repeat the preparation regimen, and adverse events associated with each regimen.

## Methods

### Study design

**Fig. 1:**
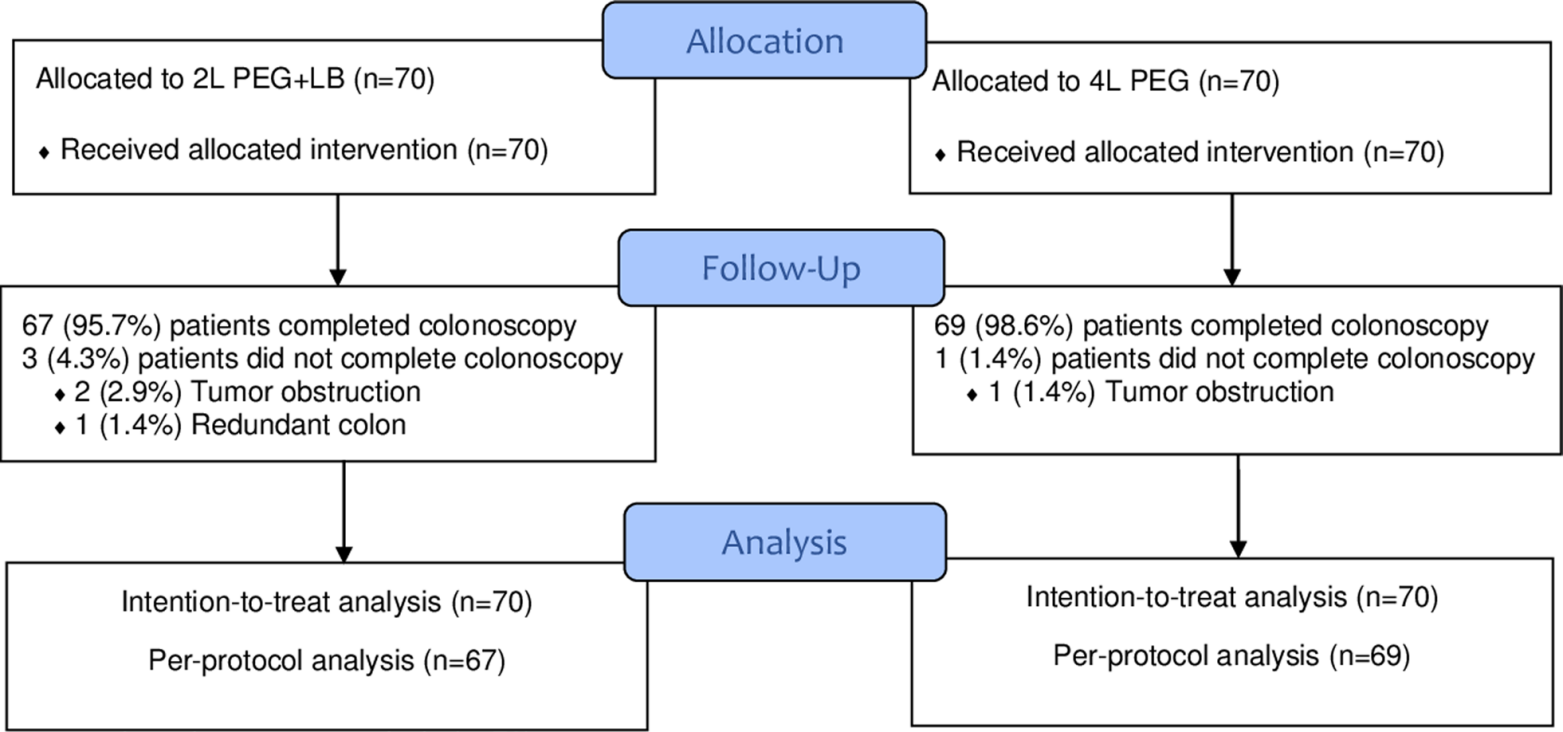
Study flow diagram. PEG, Polyethylene glycol; LB, Lubiprostone

This was a single-center, prospective, outcome assessor-blinded, randomized controlled trial (RCT) to compare the quality of bowel preparation using 2 L PEG + LB versus 4 L PEG. It was conducted between December 1, 2019 and June 30, 2021 at the Department of Medicine, Rajavithi Hospital, a tertiary referral center in Bangkok, Thailand. The protocol was reviewed and approved by the ethics committee of Rajavithi Hospital and the RCT was registered with www.clinicaltrials.gov (registration date 24/10/2019, NCT04138004). Study enrollment, eligibility screening, randomization, and completion were conducted according to the Consolidated Standards of Reporting Trials (CONSORT) guidelines (Fig. 1). The study was carried out in accordance with the ethical principles of the Declaration of Helsinki and written informed consent was obtained from the patients prior to study enrollment.

### Participants

All patients aged 18–75 years with an appropriate indication for elective colonoscopy were eligible. We excluded patients (1) with suspected gastric outlet or bowel obstruction; (2) with a history of gastrointestinal surgery apart from appendectomy or cholecystectomy; (3) with severe cardiac or pulmonary disease (American Society of Anesthesiologists physical status class 3 or 4), severe renal failure (creatinine clearance < 30 mL/min), decompensated liver cirrhosis, or severe systemic illness; (4) with a compromised swallowing reflex or impaired mental status; (5) who were pregnant or lactating; or (6) who were hypersensitive or allergic to PEG or LB. Medications that affect gastrointestinal motility and laxatives were discontinued one week before the beginning of the study.

### Randomization and blinding

All patients attended an informational session before the colonoscopy at which they were counseled about the nature and protocols of the study. They were randomly assigned to receive either 2 L PEG + LB or 4 L PEG, with an allocation ratio of 1:1. A qualified statistician used a computer to generate a randomization table with a block size of eight. Concealment was maintained by using consecutively numbered sealed envelopes. An endoscopy nurse assigned patients to their group and instructed them on the proper performance of their assigned bowel preparation method and provided them with dietary advice. The endoscopists who performed the outcome assessment and investigators were blinded to group allocation, and they were not allowed to perform any activities associated with the bowel preparation and avoided any discussion with the patients and the nurse that might lead to disclosure of the type of bowel preparation used.

### Bowel preparation method

The PEG used in the study was Niflec® (Ajinomoto Pharmaceuticals, Tokyo, Japan), which comprises PEG (macrogol) 4000 plus electrolytes (sodium sulfate, sodium hydrogen carbonate, sodium chloride, and potassium chloride). It is taken after diluting one sachet with 2 L plain water. The patients were instructed to take 250 mL every 15 min until the entire solution was consumed (1 L per hour). In the 2 L PEG + LB group, 1 L preparation administration started in the evening of the pre-procedure day at about 8.00 to 9.00 pm and the remaining 1 L was administered in the morning at about 5.00–6.00 am on the procedure day. Additionally, one tablet of 24 µg LB (Amitiza®, Catalent Pharma Solutions, St. Petersburg, Florida, USA) was given two hours before PEG ingestion (at 6.00 pm on the pre-procedure day). In the 4 L PEG group, 2 L preparation administration started in the evening of the pre-procedure day at about 8.00–10.00 pm and the remaining 2 L was administered in the morning at about 5.00–7.00 am on the procedure day.

All patients were advised to consume a low-residue diet with consumption of fruit, legumes, and vegetables forbidden from two days before the procedure. On the day before the colonoscopy, the patients had a light breakfast and lunch, but a liquid dinner (clear soup). From midnight before the procedure day, all patients were instructed to follow nil per oral (NPO) guidelines, though anti-hypertensive drugs and minimal plain water were permitted.

### Colonoscopy

All colonoscopies were performed by three experienced gastroenterologists (minimum experience of 1000 procedures) at Rajavithi Hospital, using video colonoscopes (CF 180, Olympus, Tokyo, Japan) with the patients under moderate intravenous sedation. The colon was categorized as left colon (rectum to splenic flexure), transverse colon (splenic to hepatic flexure), and right colon (hepatic flexure to cecum).

An incomplete colonoscopy was defined as an endoscopy in which the endoscope could not reach the ileocecal valve and cecum. Procedure time was the period from the endoscope entering the anus to it being withdrawn. Images were recorded during the whole procedure, and when the procedure was finished, the bowel preparation quality was assessed.

### Outcome measurement

The primary endpoint was colon cleanliness, which was evaluated by three endoscopists (AS, KC, and TC) who were blind to the bowel preparation regimen, using the validated Boston Bowel Preparation Scale (BBPS) score [11, 12]. The images of each colonic segment (right, transverse, and left colon segments) were reviewed and graded from 0 (solid stools) to 3 (no residual staining), and then summed for a total score of 0–9. The maximum BBPS score (9) represents perfect cleanliness, and the minimum BBPS score (0) represents an unprepared colon. The rate of adequate bowel preparation, which was defined as a total BBPS score ≥ 6 and/or all segment scores ≥ 2 [13], was also assessed. Before the study began, the endoscopists were given a presentation on bowel preparation images and BBPS scoring, and a calibration exercise was performed.

The secondary outcomes were cecal intubation rate, procedure time, withdrawal time, polyp detection rate, adenoma detection rate, patient satisfaction (based on a 10-cm visual analog scale; 10 indicates excellent and 0 indicates very bad), compliance (based on whether the patient completely or incompletely ingested all the assigned PEG solution), willingness to repeat the preparation regimen in a future colonoscopy, and adverse events associated with the bowel preparation such as nausea, vomiting, bloating, abdominal pain, and dizziness. Compliance, willingness to repeat the preparation regimen, and adverse events were assessed based on an endoscopy nurse interviewing the patients and completing an associated questionnaire on the morning prior to the colonoscopy.

### Sample size calculation

The sample size was calculated using a formula for research involving two independent proportions. The calculation used data from a previous study in 2008 [14], in which the group that received single-dose 24 µg LB rather than placebo prior to split-dose 4 L PEG had a higher percentage of patients with adequate bowel preparation (86% versus 56%). The minimum required number of participants was calculated to be 35 per group for power of 80% with an alpha value of 5%.

### Statistical analysis

Statistical analyses were performed using SPSS software version 17.0 (SPSS Inc., Chicago, IL, USA). The primary outcomes (BBPS scores and rate of adequate bowel preparation) were compared between the 2 L PEG + LB and 4 L PEG groups using both intention-to-treat (ITT) and per-protocol (PP) analyses (the latter taking into account incomplete colonoscopy due to factors such as tumor obstruction). The secondary outcomes (cecal intubation rate, procedure time, withdrawal time, polyp detection rate, adenoma detection rate, patient satisfaction, compliance, willingness to repeat the preparation regimen, and adverse events) were compared between the two groups using Wilcoxon Rank Sum test. Categorical variables were compared using the chi-square test or Fisher’s exact test, as appropriate. Continuous variables were compared using the independent-samples *t*-test or the Mann–Whitney *U* test, as appropriate. All statistical comparisons were two-tailed and a *p*-value < 0.05 was considered statistically significant.

## Results

### Patient demographics, clinical characteristics, and endoscopic findings

Between December 1, 2019 and June 30, 2021, 767 outpatient colonoscopies were completed at the Department of Medicine, Rajavithi Hospital. Of these, 627 were excluded from the present study. Therefore, 140 patients were randomized into two groups of 70 each (Fig. 1). Incomplete colonoscopy occurred in three patients in the 2 L PEG + LB group (two due to tumor obstruction and one due to a redundant colon) and one patient in the 4 L PEG group (due to tumor obstruction).

The mean age of the patients was 58.7 years. The majority were males (60%), while the average body mass index was 23.3 kg/m^2^. One-third of the patients had undergone a previous colonoscopy, and around 20% used laxatives. The most frequent indication for colonoscopy was colorectal cancer screening (32.2%). There were no significant differences between the groups in demographic or clinical characteristics (Table 1).

**Table 1:** Demographic and clinical characteristics of patients

Variable	Total (N = 140)	2 L PEG + LB (n = 70)	4 L PEG (n = 70)	*p*-value
Age, mean (SD)	58.7 (10.8)	58.8 (11.2)	58.5 (10.6)	0.85
Male sex, n (%)	84 (60.0)	44 (62.9)	40 (57.1)	0.49
BMI (kg/m^2^), mean (SD)	23.3 (4.2)	22.6 (4.1)	23.9 (4.3)	0.08
Education level, n (%)				0.20
None	3 (2.1)	3 (4.3)	0 (0)	
Below Bachelor’s degree	92 (65.7)	44 (62.9)	48 (68.6)	
Bachelor’s degree or higher	45 (32.2)	23 (32.8)	22 (31.4)	
Underlying diseases, n (%)				
None	46 (32.9)	22 (31.4)	24 (34.3)	0.70
Hypertension	43 (30.7)	21 (30.0)	22 (31.4)	0.86
Dyslipidemia	29 (20.7)	15 (21.4)	14 (20.0)	0.84
Diabetes mellitus	24 (17.1)	11 (15.7)	13 (18.6)	0.65
Cardiovascular	11 (7.9)	8 (11.4)	3 (4.3)	0.12
Other	20 (14.3)	9 (12.9)	11 (15.7)	0.63
Laxative use, n (%)	32 (22.9)	17 (24.3)	15 (21.4)	0.69
Previous colonoscopy, n (%)	53 (37.9)	25 (35.7)	28 (40.0)	0.60
Indication for colonoscopy, n (%)				
Colorectal cancer screening	45 (32.2)	23 (32.9)	22 (31.4)	0.86
Abdominal pain	27 (19.3)	10 (14.3)	17 (24.3)	0.13
Bowel habit change	15 (10.7)	11 (15.7)	4 (5.7)	0.10
History of colonic polyps	12 (8.6)	7 (10.0)	5 (7.2)	0.55
Lower GI bleeding	12 (8.6)	4 (5.7)	8 (11.4)	0.22
Chronic diarrhea	9 (6.4)	5 (7.1)	4 (5.7)	0.73
Iron deficiency anemia	8 (5.7)	3 (4.3)	5 (7.2)	0.72
Significant weight loss	5 (3.6)	4 (5.7)	1 (1.4)	0.37
Positive fecal occult blood test	3 (2.1)	0 (0)	3 (4.3)	0.25
Abnormal CT finding	2 (1.4)	2 (2.9)	0 (0)	0.50
Other	2 (1.4)	1 (1.4)	1 (1.4)	0.99

**Fig. 2:**
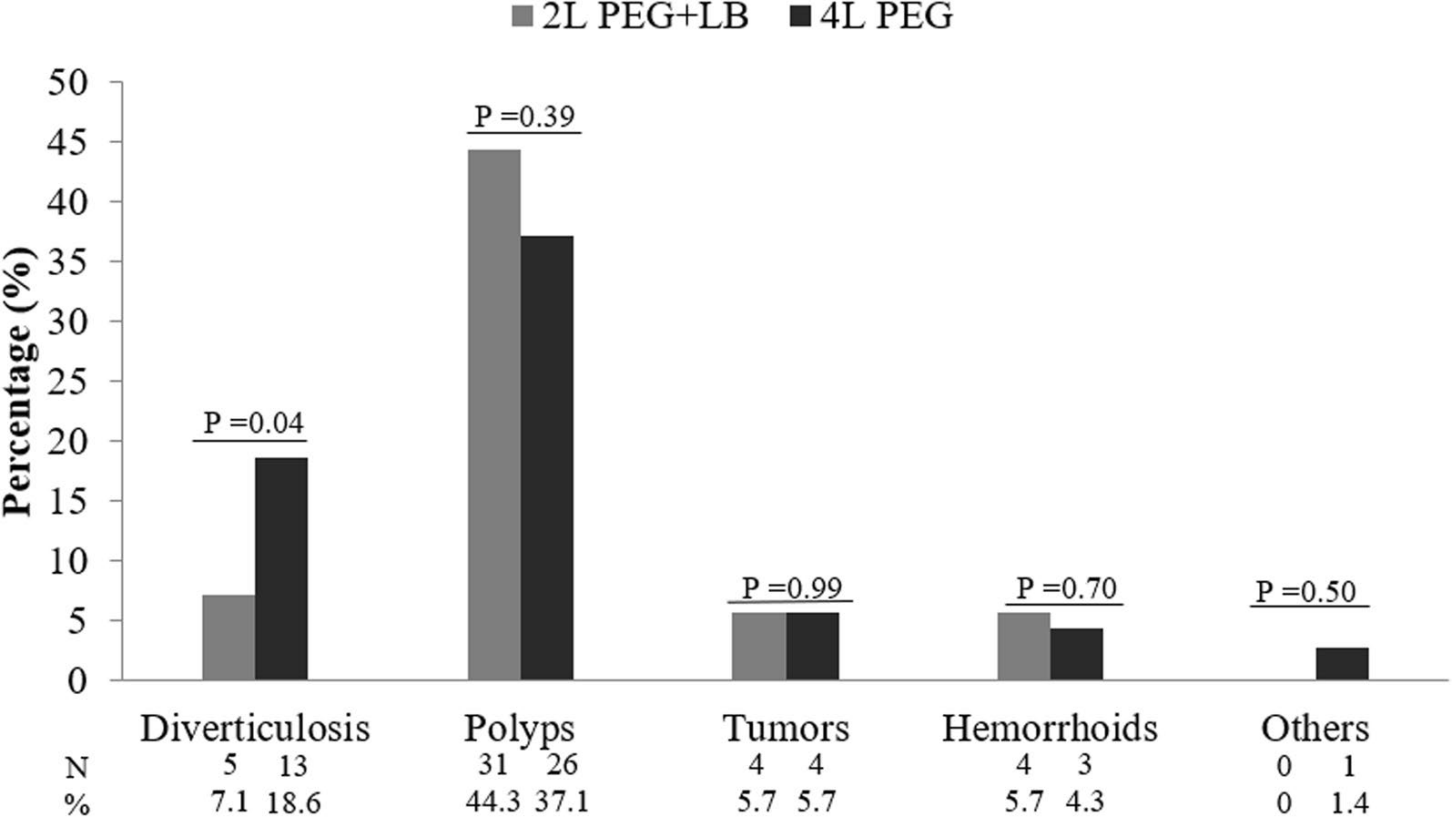
Comparison of endoscopic findings between the two groups. PEG, Polyethylene glycol; LB, Lubiprostone

Endoscopic findings were similar in the two groups except for colonic diverticulosis, which was significantly lower in the 2 L PEG + LB group than the 4 L PEG group (7.1% versus 18.6%, *p* = 0.04). The most common finding was colonic polyps, and the polyp detection rate was comparable in the two groups (44.3% versus 37.1%, *p* = 0.39) (Fig. 2). As well as, the adenoma detection rate was not significantly different between the 2 L PEG + LB group and the 4 L PEG group (34.3% versus 32.9%, *p* = 0.86) (Table 3).

### Colon cleanliness and rate of adequate bowel preparation

**Fig. 3:**
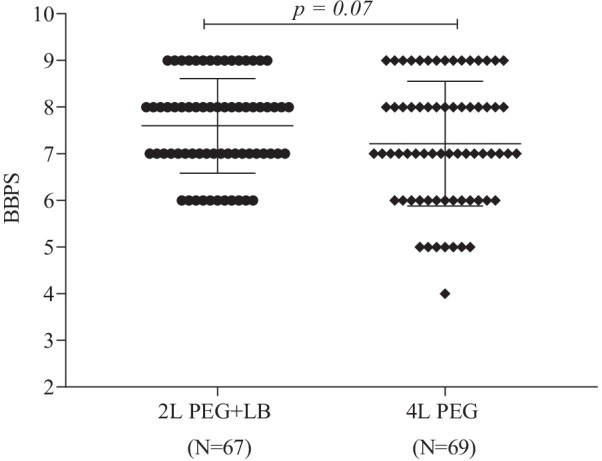
Comparison of total BBPS score between the two groups by PP analysis. BBPS, Boston Bowel Preparation Scale; PEG, Polyethylene glycol; LB, Lubiprostone; PP, per-protocol

The distribution of the total BBPS score in each group is shown in Fig. 3, and appears to be relatively consistent between groups. The mean total and segment-specific BBPS scores were not significantly different between groups in either the PP or the ITT analysis (Table 2).

**Table 2:** Colon cleanliness and rate of adequate bowel preparation

Variable	2 L PEG + LB	4 L PEG	*p*-value
BBPS score, mean (SD)			
ITT analysis	n = 70	n = 70	
Right colon	2.2 (0.7)	2.3 (0.7)	0.73
Transverse colon	2.4 (0.7)	2.4 (0.6)	0.63
Left colon	2.7 (0.5)	2.5 (0.6)	0.14
Total	7.1 (1.5)	7.4 (1.4)	0.32
PP analysis	n = 67	n = 69	
Right colon	2.7 (0.5)	2.3 (0.7)	0.30
Transverse colon	2.6 (0.5)	2.4 (0.6)	0.17
Left colon	2.7 (0.5)	2.5 (0.6)	0.14
Total	7.6 (1.02)	7.2 (1.3)	0.07
Adequate bowel preparation			
ITT analysis	n = 70	n = 70	
Percentage of patients (95% CI)	95.7 (88.0–99.1)	87.1 (77.0–93.9)	0.07
PP analysis	n = 67	n = 69	
Percentage of patients (95% CI)	100 (94.6–100)	88.4 (78.4–94.9)	0.004*

^*^P < 0.05

In the PP analysis, the rate of adequate bowel preparation was significantly higher in the 2 L PEG + LB group than the 4 L PEG group (100% [95% CI 94.6–100] versus 88.4% [95% CI 78.4–94.9], *p* = 0.004). However, in the ITT analysis, it was not significantly different (Table 2).

### Secondary outcomes

The cecal intubation rate, procedure time, withdrawal time, and satisfaction score were not significantly different between the groups. There was a non-significantly higher rate of willingness to repeat the preparation regimen in the 2 L PEG + LB group than the 4 L PEG group (94% versus 88.6%, *p* = 0.37). Regarding patient compliance, nearly all patients in the two groups completely ingested all the assigned PEG solution (100% versus 97.1%, *p* = 0.50) (Table 3). The two cases of incomplete ingestion in the 4 L PEG group were due to bloating and vomiting. Adverse events (including nausea, bloating, dizziness, and vomiting) were found in about one-fourth of patients in both groups, which was not significantly different (Table 3).

**Table 3:** Secondary outcomes

Variable	Total (N = 140)	2 L PEG + LB (n = 70)	4 L PEG (n = 70)	*p*-value
Cecal intubation rate, n (%)	136 (97.1)	67 (95.7)	69 (98.6)	0.21
Procedure time (min), median (IQR)	27 (20–35)	25 (20–35)	28.5 (20–35)	0.52
Withdrawal time (min), median (IQR)	13.5 (10–20)	14 (9–18)	13 (10–20)	0.66
Adenoma detection rate, n (%)	47 (33.6)	24 (34.3)	23 (32.9)	0.86
SSP detection rate, n (%)	6 (4.3)	3 (4.3)	3 (4.3)	0.99
HP detection rate, n (%)	24 (17.1)	9 (12.9)	15 (21.4)	0.18
^†^Proximal adenoma detection rate, n (%)	19 (13.6)	11 (15.7)	8 (11.4)	0.46
^‡^Distal adenoma detection rate, n (%)	36 (25.7)	15 (21.4)	21 (30)	0.25
Satisfaction score, median (IQR)	9 (8–10)	9 (8–10)	9 (8–10)	0.08
Satisfaction score > 8, n (%)	124 (88.6)	64 (91.4)	60 (85.7)	0.29
Compliance (based on complete ingestion of bowel preparation regimen), n (%)	138 (98.6)	70 (100)	68 (97.1)	0.50
Willingness to repeat the preparation regimen, n (%)	128 (91)	66 (94)	62 (88.6)	0.37
Adverse events, n (%)				0.89
None	102 (72.8)	50 (71.4)	52 (74.3)	0.70
Nausea	28 (20.0)	16 (22.8)	12 (17.1)	0.40
Bloating	5 (3.6)	2 (2.9)	3 (4.3)	0.65
Dizziness	4 (2.9)	2 (2.9)	2 (2.9)	0.99
Vomiting	1 (0.7)	0 (0)	1 (1.4)	0.32

SSP, sessile serrated polyp; HP, hyperplastic polyp

^†^Proximal to the splenic flexure

^‡^Distal to the splenic flexure

## Discussion

Good bowel preparation is a critical determinant of a successful colonoscopy, and is related to the safety, diagnostic accuracy, and speed of the examination [1]. The adenoma missed rate in patients with inadequate bowel preparation on initial screening colonoscopy was reported to be 42.4–47.9% [4, 15] and was an important predictor of colorectal cancer occurrence after colonoscopy [1]. Standard bowel preparation regimes have not been established.

This study assessed the colon cleanliness and the adequacy of bowel preparation with LB pretreatment plus a low-volume (2 L) split-dose PEG preparation regimen for colonoscopy. We found that 2 L PEG + LB was as good as 4 L PEG (with no significant differences) regarding bowel cleansing efficacy based on the mean total and segment-specific BBPS scores, including for the right colon. Moreover, the rate of patients with adequate bowel preparation was significantly higher in the former compared to the latter group in the PP analysis. These results imply that LB may be a beneficial adjunct to decrease the volume of PEG required for optimal bowel preparation.

Previous studies have reported that the rate of adequate bowel preparation with 4 L PEG regimen ranged from 71.3 to 92.1% [16]. In the present study, it was 88.4%, and it increased to 100% with the addition of 24 µg LB to 2 L PEG. LB is a novel dual-action laxative with both a cathartic effect and a positive effect on intestinal transit time (increasing the frequency of bowel movements) [17]. These effects may increase the bowel purgative efficiency of PEG. This was supported by a recent meta-analysis of five RCTs, which evaluated LB as a bowel cleansing agent in combination with PEG [18]. Similar to our findings, the addition of LB to the PEG bowel preparation regimen significantly increased the rate of patients with excellent preparation (RR: 1.68 [95% CI 1.40–2.02], *p* < 0.00001) and there was a trend towards a decreased rate of patients with poor preparation (RR: 0.57 [95% CI 0.30–1.08], *p* = 0.09).

In addition, the adenoma detection rate (ADR), one of the benchmarks of adequate detection at screening or diagnostic colonoscopy, in our study was also satisfactory (34.3% in the 2 L PEG + LB group and 32.9% in the 4 L PEG group). The ADR in the 2 L PEG + LB group reached the minimum standard (for primary screening colonoscopy) according to the European guidelines that recommend that ADR should be ≥ 25% [19]. Therefore, our results ensure that 2 L PEG + LB meets important performance criteria for lower gastrointestinal endoscopy (e.g., adequate bowel preparation rate ≥ 90% and ADR ≥ 25%) [19], suggesting that this regimen is suitable for both screening and routine clinical colonoscopies.

Furthermore, a factor that may have contributed to outstanding bowel preparation in our study is that the patients were advised to consume a low-residue diet for two days prior to the procedure. This is in contrast to a study by Stengel et al. [14] that compared single-dose 24 µg LB to placebo prior to 4 L split-dose PEG bowel preparation and did not involve dietary restriction. In the study [14], the LB group had a lower rate of poor bowel preparation (14%) than the placebo group (44%), which is considerably higher than the rates in our study (0% in the 2 L PEG + LB group and 11.6% in the 4 L PEG group by PP analysis). Thus, our results reflect that a low-residue diet before colonoscopy coupled with effective laxatives is more likely to lead to a higher rate of adequate bowel preparation.

Despite the high volume, 4 L PEG is the most commonly used bowel preparation regimen, and it has a well-established safety profile. However, the large fluid intake and adverse events, including nausea and vomiting, represent major barriers to patient compliance and are associated with inadequate bowel preparation [16]. To reduce the volume of PEG in order to improve tolerability, low-volume (2 L) PEG plus osmotically active ascorbic acid has been used. A meta-analysis [20], involving 11 RCTs comparing 2 L PEG plus ascorbic acid versus 4 L PEG as bowel preparations for colonoscopy, showed a non-inferior bowel cleansing efficacy (OR: 1.08 [95% CI 0.98–1.28], *p* = 0.34) but significantly better compliance for 2 L PEG plus ascorbic acid (OR: 2.23 [95% CI 1.67–2.98], *p* < 0.00001), with reduced nausea and vomiting. Nonetheless, PEG plus ascorbic acid is not recommended in patients with severe renal insufficiency, congestive heart failure, phenylketonuria, or glucose-6-phosphate dehydrogenase deficiency [21]. Other adjuncts, including bisacodyl and magnesium citrate, lead to similar outcomes to ascorbic acid, but high-dose bisacodyl can cause ischemic colitis [8] and magnesium citrate is not recommended in patients with severe renal insufficiency or congestive heart failure. [21]

Contrary to our predictions, patient compliance (i.e., complete ingestion of the assigned preparation regimen) with the 4 L PEG regimen was very good, which may be explained by the educational session about the bowel preparation methods provided before the colonoscopy and the study environment. Compliance did not differ between the 2 L PEG + LB group and the 4 L PEG group (100% versus 97.1%, *p* = 0.50), although both the compliance and the willingness to repeat the preparation regimen (94% versus 88.6%, *p* = 0.37) were non-significantly higher in the 2 L PEG + LB group. Additionally, there were no differences in adverse events between the groups (Table 3). Nevertheless, the results were in line with our hypothesis that LB can be used as an adjunct to a low-volume (2 L) split-dose PEG regimen, which has the advantage of being easy to consume and is a desirable regimen as it has excellent bowel cleansing efficiency and a good safety profile.

This is the first study to demonstrate that reducing the amount of split-dose PEG from 4 to 2 L still leads to satisfactory bowel preparation when it is combined with LB. Our 2 L PEG + LB regimen remained similar to the standard recommended method and general practice of using a split-dose bowel preparation regimen prior to most elective colonoscopies performed in the morning.

The study has some limitations. It was a single-center study that only recruited outpatients. The exclusion of hospitalized patients (who are more likely to have serious co-morbidities and to be immobilized) may have inadvertently excluded cases involving more challenges to bowel cleansing. The results may have also been affected by the dietary restriction, as many Thai foods contain fruits and vegetables, so the results may not be generalizable to bowel preparation regimens that do not incorporate dietary restriction. We believe that dietary restriction should be recommended prior to colonoscopy.

In conclusion, the addition of LB pretreatment to low-volume (2 L) split-dose PEG leads to bowel cleansing efficacy that is equivalent, in terms of BBPS scores, to that of high-volume (4 L) split-dose PEG. Additional studies in different populations are needed prior to adding LB to standard PEG bowel preparation regimens.

## Data Availability

The datasets generated during and analyzed during the current study are not publicly available due to consent for publication of raw data not obtained and the dataset could, in theory, pose a threat to the confidentiality of the study participants but are available from the corresponding author on reasonable request.

## Abbreviations

PEG: Polyethylene glycol
LB: Lubiprostone
BBPS: Boston Bowel Preparation Scale
SSP: Sessile serrated polyp
HP: Hyperplastic polyp
